# Fabrication of functional micro–nano structures using phase holographically modulated femtosecond laser technology: a review

**DOI:** 10.1515/nanoph-2025-0131

**Published:** 2025-09-04

**Authors:** Yang Liu, Jie Wang, Zhipeng Wang, Ji Huang, Xuejiao Wang, Jingjing Zhang, Zijie Dai, Yunxia Ye

**Affiliations:** School of Mechanical Engineering, 12676 Jiangsu University, Zhenjiang, 212013, China; Laser Micro/Nano Fabrication Laboratory, School of Mechanical Engineering, Beijing Institute of Technology, Beijing, 100081, China; National Institute of Metrology, Beijing, 100029, China

**Keywords:** phase hologram, spatial modulation, femtosecond laser, laser fabrication, functional micro–nano structure

## Abstract

Micro–nano fabrication technology is critical to high-end fabrication, bridging the gap between microscopic and macroscopic scales. Femtosecond laser fabrication, owing to its ultrafast nonlinear effects and three-dimensional direct writing capabilities, has demonstrated unique advantages in the fabrication of functional micro–nano structures. Phase holographically modulated femtosecond laser technology, as a representative of spatial optical field modulation, modulates the phase of the incident laser field to flexibly transform a single focal point into various spatial optical fields, including multifocal arrays, patterned optical fields, and three-dimensional optical fields, according to specific fabrication requirements. This technology not only improves fabrication precision and efficiency but also provides greater flexibility in femtosecond laser fabrication. This review systematically summarizes recent technological advances, focusing on four aspects: algorithms for generating phase holograms, exceeding the diffraction limit to improve fabrication resolution, optimizing fabrication quality, and improving fabrication efficiency. It aims to provide theoretical foundations and technical references to support the practical application of modulated femtosecond laser technology in the fabrication of functional micro–nano structures.

## 1 Introduction

With the development of society and economy, along with the increasing demand for functionalization, there is a growing requirement for higher processing quality, miniaturization, and integration. This trend presents new opportunities and challenges for fabricating technologies, particularly high-end manufacturing [1], [2]. Functional micro–nano structures (FMNS) exhibit unique properties in various fields, including optics [3], [4], [5], [6], [7], mechanics [8], [9], [10], [11], [12], [13], energy [14], [15], [16], [17], [18], [19], biomimetics [20], [21], [22], [23], [24], and sensing [25], [26], [27], [28], [29], [30], making the exploration of new FMNS fabrication technologies a widely studied research focus worldwide. The fabrication of FMNS typically involves small-scale, highly customized processing, where their performance is significantly influenced by morphological accuracy and surface defects [31]. Therefore, balancing quality, efficiency, and cost is a critical problem in the FMNS manufacturing process [32].

**Figure 1:**
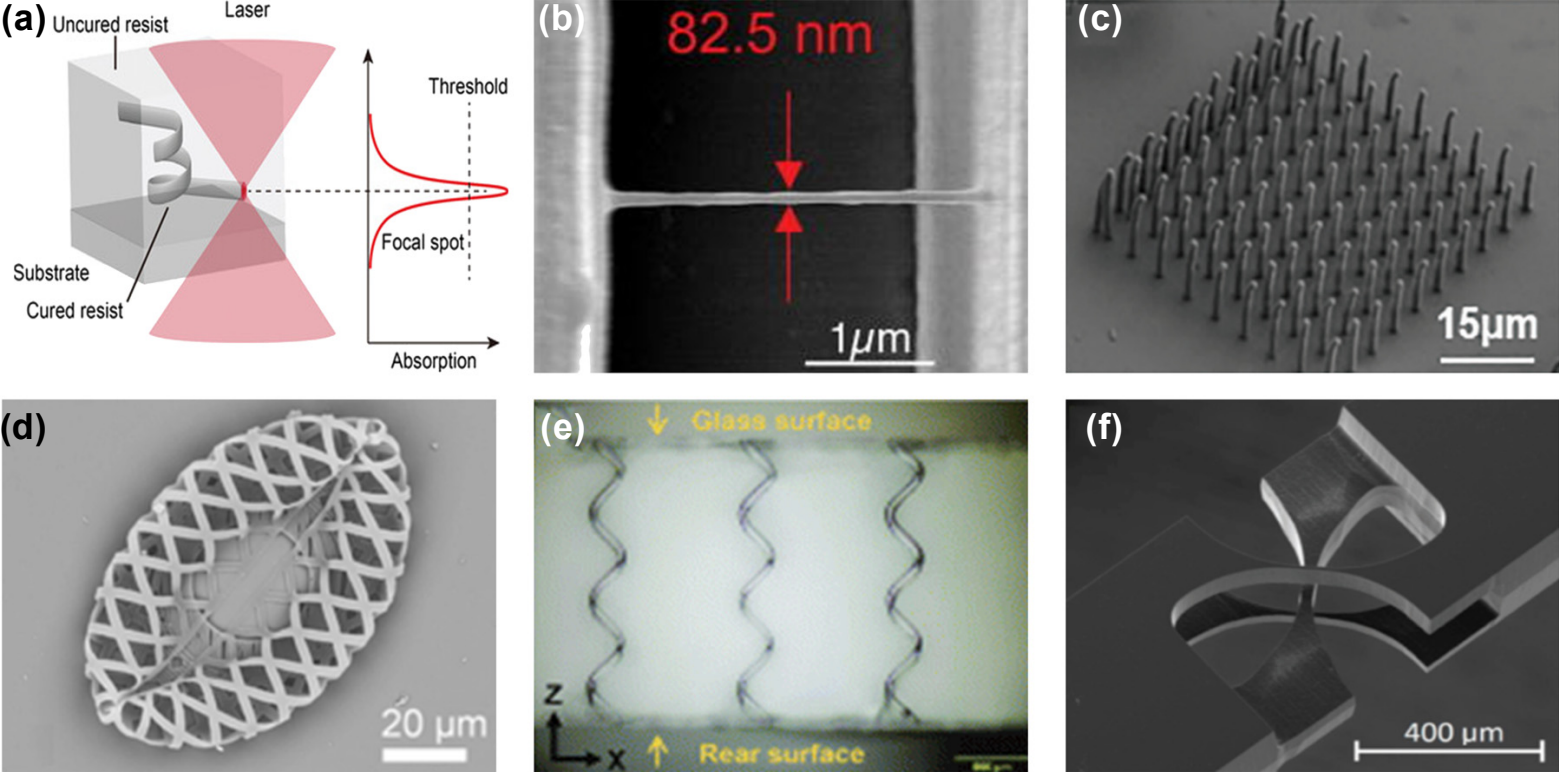
Subdiffraction-limit FMNS and 3D FMNS using the threshold and nonlinear effects of femtosecond lasers. (a) Schematic of the nonlinear multiphoton absorption and threshold effects induced by a femtosecond laser [9]. (b) Subdiffraction-limit structures fabricated via femtosecond laser TPP [49]. (c) 3D structures fabricated using femtosecond laser TPP [50]. (d) Metal-photoresist composite 3D structures fabricated via femtosecond laser-induced two-photon reduction/polymerization [51]. (e) Microchannel structures fabricated by femtosecond laser ablation inside glass [52]. (f) Femtosecond laser modification-assisted hydrofluoric acid (HF) etching was used to fabricate a fused silica cross-shaped elastic hinge [53]. (a) Is reprinted from Ref. [9], with permission. Copyright 2023 John Wiley and Sons; (b) is reprinted from Ref. [49], under the terms of the Open Access Publishing Agreement; (c) is reprinted from Ref. [50], with permission. Copyright 2024 John Wiley and Sons; (d) is reprinted from Ref. [51], with permission (CC BY 4.0); (e) is reprinted from Ref. [52], with permission. Copyright 2022 Royal Society of Chemistry; (f) is reprinted from Ref. [53], under the terms of the Open Access Publishing Agreement.

Femtosecond lasers have provided a powerful, noncontact, mask-free, high-precision, and environmentally friendly fabrication method capable of arbitrary patterning for the fabrication of FMNS, offering an effective solution to the challenges above [33]. The femtosecond-scale pulse duration enables the peak power density of a focused femtosecond laser to reach up to 10^22^ W/cm^2^. Compared with conventional continuous-wave and long-pulse lasers, femtosecond lasers exhibit ultrafast and ultra-intense characteristics [34], [35], [36], leading to high processing quality [37], [38], [39], [40], [41], [42] and broad material adaptability [43], [44], [45], [46] in femtosecond laser fabrication. In addition, femtosecond lasers can exceed the optical diffraction limit due to their nonlinear ionization mechanism, achieving nanoscale processing precision [47]. As illustrated in Figure 1a, nonlinear ionization and material modulation are induced only when the energy density of the femtosecond laser focal spot exceeds a specific threshold [9], [48]. Therefore, by precisely controlling the energy of the femtosecond laser, the energy density at the focal spot can be confined to just above the nonlinear ionization threshold, enabling subdiffraction-limit processing resolution (defined as the minimum distance between two adjacent features) in FMNS fabrication, as illustrated in Figure 1b [49]. Moreover, femtosecond lasers possess the capability for three-dimensional (3D) fabrication within transparent materials due to the aforementioned nonlinear effects. By focusing the laser beam inside the material, the nonlinear ionization threshold effect ensures that polymerization [50], reduction [51], removal [52], or modification [53] occurs only within the focal region, as illustrated in Figure 1c–f. Furthermore, arbitrary 3D structures can be precisely fabricated by integrating specific laser scanning paths [54].

Femtosecond laser fabrication can be broadly classified into two approaches: subtractive fabrication and additive fabrication. Owing to the ability of femtosecond lasers to achieve high photon densities in both spatial and temporal domains, they are well-suited for high-quality subtractive fabrication of any material via multiphoton absorption. This includes direct laser writing for layer-by-layer material removal, or laser-induced modification followed by chemical etching. In contrast, additive fabrication, exemplified by two-photon polymerization (TPP), also relies on the nonlinear effect of two-photon absorption to construct 3D structures. TPP imposes strict requirements on both material properties and fabrication conditions; as a result, it is generally limited to materials with narrow bandgaps, such as thermoplastics, photopolymers, and a few specialized types of glass [55]. For hard materials with high thermal stability and mechanical strength, subtractive fabrication remains the preferred approach.

Although femtosecond laser micro–nano fabricating technology has been successfully applied to the fabrication of various FMNS, conventional femtosecond laser fabricating still typically relies on a single-focus direct writing approach. This approach becomes highly time-consuming when fabricating large-scale or complex FMNS, significantly limiting the practical application of femtosecond laser micro–nano fabricating technology.

To improve fabrication efficiency, a high-speed galvanometer was often integrated into the femtosecond laser fabricating system to increase the scanning speed of the laser focal point [56], [57], [58]. However, galvanometer scanning generally requires specialized F-theta lenses for beam focusing. The focusing capability of F-theta lenses is lower than that of high numerical aperture (NA) objective lenses, resulting in a focal spot size typically in the range of tens of micrometers, which is insufficient for achieving sub-micrometer or nanoscale fabricating resolution. A high-NA objective lens can be used instead of an F-theta lens in combination with a galvanometric scanner to compensate for the scanner’s limited focusing capability. However, due to the typically small entrance aperture of high-NA objectives, the beam reflected from the scanner usually needs to be demagnified through a 4*f* system before reaching the objective lens, resulting in a more complex optical setup. An alternative and effective strategy for improving femtosecond laser fabrication efficiency is spatial optical field modulation. During the interaction between femtosecond lasers and materials, electrons act as carriers that absorb photon energy, and the nature of electronic excitation determines the material’s subsequent phase transition pathway. If a single femtosecond pulse is temporally divided into a series of subpulses (with adjustable energy ratios between them), the excitation states of electrons can be manipulated. [43]. Meanwhile, by spatially modulating the femtosecond laser through parameters such as field intensity, phase, and polarization direction, the photon–electron interaction process can also be controlled, thereby influencing the local transient electron density, temperature, and excitation states. Consequently, spatial modulation of the optical field enables localized and controllable modulation of the material’s transient physicochemical properties, as well as its subsequent phase transitions and structural formation processes. Whether in additive or subtractive fabrication, spatial optical field modulating can significantly enhance the resolution, quality, and efficiency of femtosecond laser fabrication [43].

Spatial optical field modulation methods can be classified into static and dynamic optical field modulation based on the type of optical modulation elements employed. Static optical field modulation refers to using fixed-phase or amplitude-type optical elements to modulate the incident beam, thereby generating a specific optical field distribution. For instance, microlens arrays [59], optical diffractive elements [60], and dual/multibeam interference systems [61] can be used to transform a single femtosecond laser focus into a periodically arranged or patterned multifocal points array, allowing for the simultaneous fabrication of multiple FMNS structures. Although static optical field modulation has been widely applied to enhance the efficiency of femtosecond laser fabrication, its flexibility remains inherently limited. This limitation arises from the use of fixed optical modulation elements, which can only produce predefined optical field distributions, thereby preventing the generation of arbitrary modulated optical fields or real-time dynamic modulation [62].

Dynamic adaptive optical field modulation, based on dynamic optical field modulation devices, uses addressable pixel arrays to achieve pixel-level modulation of the incident optical field. Depending on the modulation mechanism, these devices can be classified into amplitude-type and phase-type. A typical amplitude-type dynamic optical field modulation device is the digital micromirror device (DMD) [63], which controls the on–off and duration of laser reflection in specific directions by adjusting the orientation of micromirror pixel units. This enables the generation of structured multifocal arrays or patterned optical fields. The key advantage of DMD technology is its high refresh rate, reaching up to 40 kHz, and its ability to generate the desired modulated optical field without requiring complex algorithms. However, a significant limitation is its relatively low modulation efficiency, leading to low energy utilization.

In contrast, phase-type dynamic optical field modulation devices digitally modulate the phase of the incident optical field, offering significantly higher modulation efficiency. Common phase modulators include the deformable mirror (DM) [64] and the spatial light modulator (SLM) [65], [66]. DM modulates the phase of the incident laser by adjusting the propagation distance of the reflected wavefront through mirror surface deformation and making it primarily suitable for wavefront correction and optical field quality optimization. In comparison, SLM modulates the phase of the incident laser by adjusting the refractive index of liquid crystal molecules, providing higher pixel resolution and broader applicability than DM. By loading specific phase holograms onto the SLM, the control system applies specific voltages to individual liquid crystal cells according to the desired phase at each pixel. These voltage variations alter the orientation of the liquid crystal molecules, thereby changing their birefringence. Thereby, the phase of the incident beam is modulated accordingly, and theoretically enables the generation of arbitrarily distributed intensity patterns. These characteristics make SLM-based phase holographic spatial optical field modulation a critical technology in optical information processing and adaptive optics systems. It has been extensively applied in holographic imaging [67], optical tweezers [68], microscopic imaging [69], beam modulation [70], and various other fields.

Numerous scholars have extensively investigated SLM-based phase holographic spatial optical field modulation. However, no comprehensive review has been published summarizing its applications in FMNS fabrication. Given this, the article presents a systematic review, with a particular focus on this technology in four key aspects: algorithms for generating phase holograms, exceeding the diffraction limit to enhance fabrication resolution, optimizing structural uniformity, and improving fabrication efficiency. Specifically, this review focuses on the classification and distinctive features of phase hologram generation algorithms, the achievement of nanoscale feature fabrication through dual-focus interference and multimodal optical field modulation, the improvement of FMNS quality via spherical aberration compensation algorithms and noise suppression strategies, and the advancement of fabrication efficiency through multifocal parallel fabrication, image-based projection, and 3D fabrication technologies.

## 2 Phase hologram generation algorithms

In phase holographically modulated femtosecond laser technology, the generation of phase holograms is a critical step for achieving precise control of the optical field. Depending on how phase and amplitude are computed, existing methods can be broadly categorized into three types: iterative optimization methods, forward modeling approaches, and deep learning–based technologies.

### 2.1 Iterative optimization algorithm

Iterative optimization algorithm, such as the Gerchberg–Saxton (GS) algorithm [71], weighted GS (GSW) algorithm [72], mixed-region-amplitude-freedom (MRAF) algorithm [73], optimal rotation angle (ORA) algorithm [74], adaptive-additive (AA) algorithm [75], simulated annealing method [76], and the Yang–Gu algorithm [77], rely on iterative feedback loops to optimize the phase distribution. These algorithms differ in their objective functions and weighting strategies, leading to different performances in terms of optical field uniformity, energy efficiency, and computational speed. They are commonly used to construct complex structures such as multifocal arrays, planar optical fields, and volumetric optical fields. However, these methods typically require a large number of iterations to converge and must be recalculated for different target optical fields, which limits their flexibility and real-time applicability.

### 2.2 Forward modeling algorithm

The forward modeling algorithm constructs target optical fields by superposing an analytical phase function. A representative example is the zonal modulation method [78], [79], which divides the incident optical field into regions and applies different phase profiles to generate two- or three-dimensional multifocal arrays. Other frequently employed models include Dammann gratings [80], Fresnel diffraction [81], and π-phase plates [78], [82]. These approaches offer high energy utilization and are well-suited for laser processing applications that require specific intensity or polarization distributions.

### 2.3 Deep learning–based algorithm

Deep learning–based algorithms have been introduced more recently for phase hologram generation, utilizing architectures such as convolutional neural networks (CNNs) [83] and diffractive neural networks (DNNs) [84]. These data-driven methods do not rely on physical models and can directly predict phase distributions, enabling rapid generation of highly complex optical fields. Additionally, the incorporation of phase gradient terms into the loss functions can reduce speckle noise. Although these methods may incur higher computational costs, they offer advantages for applications demanding high uniformity or real-time modulation.

In summary, each type of algorithm exhibits distinct advantages in terms of accuracy, efficiency, and applicability. The choice of phase algorithm should, therefore, be made based on the specific requirements of the intended application.

## 3 Improvement of fabrication resolution: exceeding the diffraction limit in FMNS structure fabrication

Femtosecond lasers have achieved subdiffraction-limit resolution in the fabrication of dielectrics and polymer materials through nonlinear effects. However, a key challenge in the femtosecond laser micro–nano fabrication remains the realization of subwavelength or even nanoscale structures in metals and semiconductors – commonly referred to as super-resolution fabrication. This issue has long been a research focus in the field.

There are two primary approaches to achieving super-resolution fabrication with femtosecond lasers: (1) employing shorter laser wavelengths to reduce the optical diffraction limit and (2) modulating the optical field to precisely control light–material interactions, thereby breaking through the diffraction limit [85]. The former approach is costly and lacks flexibility, whereas the latter, using phase holographic modulation, allows for precise control over the energy distribution of the focused optical field. The second approach not only retains fabrication flexibility but also effectively breaks through the optical diffraction limit, facilitating the fabrication of FMNS structures with nanoscale precision.

### 3.1 Dual-focus optical field–induced material phase transition and ablation

**Figure 2:**
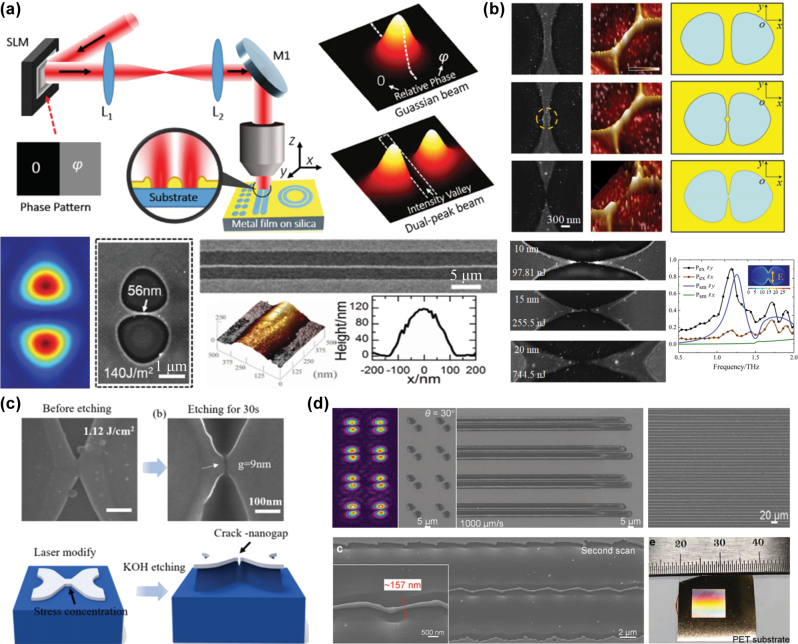
Dual-focus optical field–induced material phase transition and ablation for FMNS fabrication. (a) Gold nanowires [86], (b) gold nanoslit structures [87], and (c) crack-like silicon-based nanoslit structures [88] fabricated using a 0-π phase-modulated dual-focus femtosecond laser optical field. (d) Nanowires exceeding the optical diffraction limit were fabricated using near-field electromagnetic enhancement and incubation effects [89]. (a) Is reprinted from Ref. [86], with permission. Copyright 2015 John Wiley and Sons; (b) is reprinted from Ref. [87], with permission. Copyright 2019 American Chemical Society; (c) is reprinted with permission from Ref [88] © Optica Publishing Group; (d) is reprinted from Ref. [89], with permission. Copyright 2022 John Wiley and Sons.

Wang et al. [86] used a 0-π phase modulation to transform a Gaussian femtosecond laser beam into a dual-focus optical field with zero intensity at the center. They modulated the phase transition and localized material transfer of a gold film by controlling the spatial distribution of free electrons, ultimately fabricating gold nanowires with a minimum linewidth of 56 nm between two ablation pits, as shown in Figure 2a. Furthermore, they fabricated integrated circuits with uniform linewidths and arbitrarily arranged patterns by switching phase holograms during beam scanning using the dynamic modulation capability of the SLM. Based on this approach, Xu et al. [87] increased the laser energy to induce Rayleigh instability in the nanowires, resulting in the rupture of the nanowires and the formation of nanoslits with a minimum spacing of 30 nm. They further demonstrated the application of these nanoslits in surface-enhanced Raman scattering (SERS) and terahertz filtering, as illustrated in Figure 2b. This method was also extended to fabricate nanoslit structures on silicon surfaces. Zhou et al. [88] used a dual-focus optical field to induce modifications on the surface of crystalline silicon, forming butterfly-shaped amorphous silicon areas. During subsequent KOH etching, stress concentration at the center of these butterfly-shaped mask structures led to their rupture, producing crack-like nanoslit structures as small as 9 nm in size, as shown in Figure 2c. Compared to strategies that use specifically modulated intensity distributions to control material transfer for nanostructure fabrication, directly reducing the focal spot size below the diffraction limit offers greater universality. Zhao et al. [89] used an SLM to generate a multifocal array beam, which was focused through a low numerical aperture (NA = 0.1) objective lens. Under the influence of near-field electromagnetic enhancement and incubation effects, this method enabled the fabrication of gold nanowires with a width of approximately λ/3, reaching 1/20 of the diffraction limit, as shown in Figure 2d. Due to the long depth-of-focus characteristic of the low-NA objective, this method enables large-area fabrication on nonplanar surfaces, such as flexible PET substrates, significantly enhancing parallel fabrication efficiency by a factor of eight. Moreover, this method eliminates the need for masks or complex postprocessing steps, providing an efficient and cost-effective solution for miniaturized electronic and optoelectronic devices.

At present, this type of technology is primarily applied to the fabrication of metals and metal film materials because of their moderate ablation thresholds. When applied to materials with lower damage thresholds, such as paraffin wax or hydrogels, even the minimal energy at the periphery of the laser spot can induce ablation or modification. This leads to poor fabricating resolution and prevents the achievement of subdiffraction-limit precision. Conversely, when applied to hard and brittle materials such as glass, sapphire, or ceramics, the wide bandgap characteristics of these materials require significantly higher laser pulse energies to induce damage. However, such high pulse energies may exceed the maximum energy threshold that the SLM can withstand. As a result, this technology is currently unsuitable for fabricating wide-bandgap, hard, and brittle materials.

### 3.2 Multimodal optical fields for FMNS fabrication

**Figure 3:**
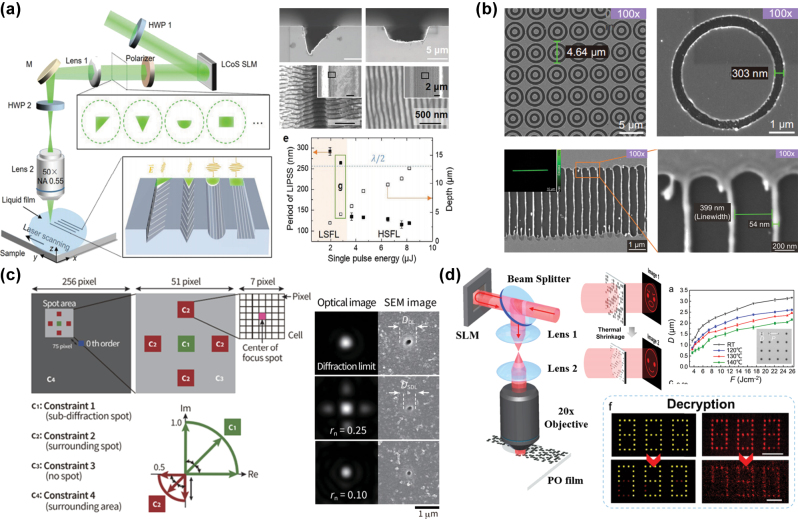
Multimodal optical fields for FMNS fabrication. (a) Schematic and SEM images of FMNS that were fabricated on the SiC surface using femtosecond laser optical field modulation combined with a liquid-assisted method [90]. (b) Annular structures and grating structures were fabricated using PPLL technology [91]. (c) Modulated focal points were achieved with a lateral size below the optical diffraction limit through interferometric cancellation between adjacent focal points [92]. (d) Micro-pit structures of various sizes fabricated on thermally shrinkable shape memory films using a modulated femtosecond laser, and the switching of the hologram from an “angry face” to a “smiling face” upon heating, as well as the decryption of “888” into “790” [93]. (a) Is reprinted from Ref. [90], with permission. Copyright 2024 John Wiley and Sons; (b) is reprinted from Ref. [91], under the terms of the Open Access Publishing Agreement; (c) is reprinted from Ref. [92], under the terms of the Open Access Publishing Agreement; (d) is reprinted from Ref. [93], with permission. Copyright 2024 American Chemical Society.

The multimodal optical field refers to an optical field capable of switching between multiple patterns. By utilizing specific effects associated with multimodal optical fields, it is possible to fabricate nanostructures with lateral dimensions below the optical diffraction limit. Qiu et al. [90] achieved simultaneous and precise control of both micron- and nanostructures in FMNS fabrication on the SiC surface by using an SLM to manipulate the optical field shape and polarization direction of the femtosecond laser, combined with micro-jet generation assisted by liquid. As shown in the SEM image in Figure 3a, the cross-sectional morphology (such as V-shaped grooves and blazed gratings) was determined by the geometric shape of the patterned laser optical field, with the characteristic width of the stripes inside the microgrooves reaching as small as 120 nm. Huang et al. [91] proposed a subwavelength-patterned pulsed laser lithography (PPLL) technology, generating quasi-binary phase masks using SLM, combined with gradient grayscale boundaries and circularly polarized light, which effectively suppressed diffraction effects and polarization asymmetry. This enabled the fabrication of high-uniformity structures, such as antennas and catenary-shaped subwavelength units on metal films and phase-change materials, with feature sizes as small as 303 nm (approximately 76 % of the optical diffraction limit), as shown in Figure 3b. Using PPLL technology, they further modulated the circular optical field into a linear form, successfully fabricating a grating with a linewidth of 54 nm. Hasegawa et al. [92] used SLM to modulate a single femtosecond laser focal point into an array of five focal points and adjusted the phase difference between the center and surrounding focal points to approximately π using algorithms. Interferometric cancellation between adjacent focal points in the array reduced the lateral size of the central focal point. The central focal spot size was reduced to 60 % of the diffraction limit by optimizing the spacing and energy ratio between the central and surrounding focal points. They used this method to fabricate microholes with diameters as small as 39 % of the optical diffraction limit on fused silica, as shown in Figure 3c. Wang et al. [93] used modulated femtosecond lasers to fabricate various sizes of micro-pit structures on polyolefin (PO) films with thermally shrinkable shape memory properties. When the PO film was heated to its glass transition temperature (∼140 °C), thermal shrinkage occurred, reducing the minimum micro-pit size to approximately 500 nm. A four-level amplitude holographic modulation was achieved using the difference in micro-pit transmittance before and after PO film shrinkage. As displayed in Figure 3d, the switching of the hologram from an “angry face” to a “smiling face” upon heating, as well as the decryption of “888”: into “790,” was experimentally verified, providing a new method for anticounterfeiting and information encryption.

Table 1 provides a summary of the fabrication resolution of FMNS achieved by different phase holographically modulated femtosecond laser technologies.

**Table 1:** Summary of fabrication resolution achieved by different phase holographically modulated femtosecond laser technologies.

Technologies	Materials	Resolution	FMNS	References
0-π phase modulation	Gold film	56 nm	Nanowire	[86]
0-π phase modulation	Gold film	30 nm	Nanogap	[87]
0-π phase modulation	Silicon	9 nm	Nanogap	[88]
Dual-peak beam combined with polarization rotation	Gold film	157 nm	Nanowire	[89]
Polarization-modulated patterned laser ablation	SiC	∼120 nm	Hierarchical microstructures	[90]
PPLL	Cr film and Au film	54 nm	Gratings	[91]
Interferometric cancellation between adjacent focal points	Quartz glass	459 nm	Microcrater	[92]
GS algorithm combined with film heat shrinkage	PO film	500 nm	Microcrater	[93]
Patterned exposure with direct laser writing	Photoresist	<100 nm	Microfluidic devices	[94]

## 4 Improvement of fabrication quality: spherical aberration compensation and uniformity optimization

### 4.1 Spherical aberration compensation

When femtosecond laser pulses are focused inside a material, the refractive index mismatch between the objective’s working medium and the material causes beam refraction at their interfaces, leading to spherical aberration at the focal point. Spherical aberration elongates the focal region axially and reduces the peak intensity, thereby influencing the fabrication quality and precision inside the material. Based on the phase modulation capability of an SLM, spherical aberration can be compensated by adjusting the wavefront of the incident beam before it enters the objective lens, thereby correcting the beam refraction at the interface and reducing focal distortion.

**Figure 4:**
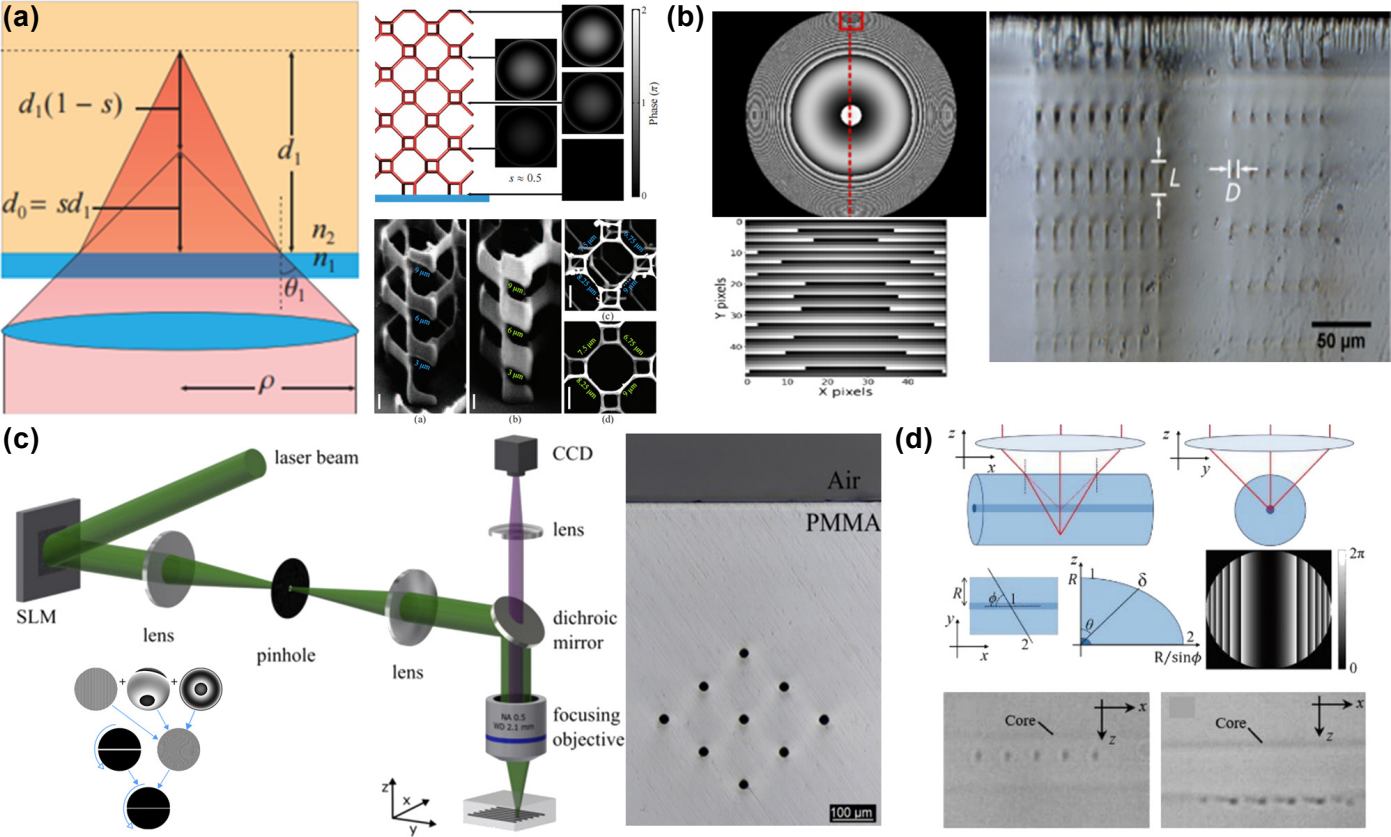
Improvement of FMNS quality through spherical aberration correction. (a) Improvement in fabrication uniformity in chiral helical photonic crystals using spherical aberration phase compensation method [95]. (b) Microstructures fabricated using the phase precompensation method [96]. (c) High uniformity microchannel fabricated in PMMA using spherical aberration phase compensation method combined with slit mask [97]. (d) Improvement in the fabrication quality of Bragg gratings inside optical fibers using spherical aberration phase compensation designed for cylindrical surfaces [98]. (a) Is reprinted from Ref. [95], under the terms of the Open Access Publishing Agreement; (b) is reprinted from Ref. [96], under the terms of the Open Access Publishing Agreement; (c) is reprinted from Ref. [97], under the terms of the Open Access Publishing Agreement; (d) is reprinted from Ref. [98], with permission (CC BY 4.0).

Cumming et al. [95] fabricated chiral helical photonic crystals inside arsenic trisulfide glass using an SLM-based spherical aberration phase compensation method. As displayed in Figure 4a, photonic crystals fabricated with spherical aberration compensation demonstrated superior morphological uniformity and agreement between experimental and simulated circular dichroism spectra compared to those without compensation. Kontenis et al. [96] further developed a phase precompensation method that dynamically adjusts the compensation phase according to fabrication depth. The microstructures fabricated by this method depth. The microstructures fabricated by this method have nearly identical axial and lateral dimensions, as shown in Figure 4b. Roth et al. [97] combined a slit mask with spherical aberration compensation phase modulation to maintain a consistently circular focal spot at different depths inside polymethyl methacrylate (PMMA), ensuring high uniformity of fabricated microchannels at various depths, as displayed in Figure 4c. Besides planar samples, spherical aberration phase compensation can also correct beam refraction when incident on curved surfaces. Salter et al. [98] analyzed the geometric characteristics of circular fiber cross sections and derived a phase compensation model for cylindrical surfaces. This model was applied to fabricate Bragg gratings inside optical fibers, as shown in Figure 4d. Compared to the uncompensated case, spherical aberration compensation significantly improved the fabrication quality of Bragg gratings, and the measured reflection spectrum showed better agreement with theoretical calculations.

### 4.2 Noise suppression for improving structural uniformity

The Gerchberg–Saxton (GS) iterative algorithm is a widely used phase compensation method [71] due to its advantages of simple algorithm flow, fast convergence speed, and high computational efficiency [99], [100]. Compared to noniterative algorithms, iterative algorithms such as the GS algorithm were able to generate structured optical fields with arbitrary shapes and intensity distributions through phase modulation of a single laser beam, offering greater versatility. However, since most iterative algorithms modulate only the amplitude distribution of the structured optical field in the focal plane, the phase distribution of the modulated optical field often exhibits randomness. This irregular phase distribution may lead to interference effects – either destructive or constructive – between adjacent focal points due to phase mismatches, thereby introducing speckle noise, degrading the uniformity of the structured optical field, and ultimately affecting the quality of FMNS fabrication.

**Figure 5:**
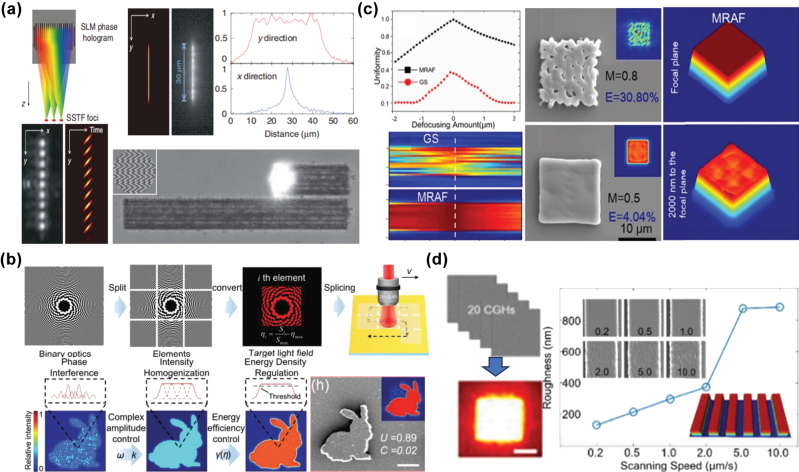
Improvement of FMNS uniformity through noise suppression. (a) Generation of a uniform line–shaped optical field and fabrication process by staggering the arrival times of each focal point at the sample via SSTF technology, effectively eliminating interference between focal points [101]. (b) Schematic and fabrication results of microstructures fabricated using a spatially modulated femtosecond laser method based on an improved algorithm [102]. (c) Improvement of structured optical field uniformity through optimization of the iterative algorithm [103]. (d) High-uniformity FMNS fabricated via dynamic scanning using the multifield noise homogenization method [94]. (a) Is reprinted from Ref. [101], with permission (CC BY 4.0); (b) is reprinted from Ref. [102], with permission, Copyright 2024 John Wiley and Sons; (c) is reprinted from Ref. [103], with permission (CC BY 4.0); (d) is reprinted with permission from Ref [94] © Optica Publishing Group.

To improve the uniformity of structured optical fields, Sun et al. [101] used a phase holography–based optical field modulation technology to generate a single-row multifocal optical field. Simultaneously, they used simultaneous spatial and temporal focusing (SSTF) technology to stagger the arrival times of each focal point at the sample, effectively eliminating interference between them. A continuous and uniform line–shaped optical field was achieved by further reducing the spacing between focal points, as shown in Figure 5a. Taking a different approach, Li et al. [102] divided the modulated optical field into a signal area and a noise area. In the signal area, phase modulation was employed to eliminate speckle noise caused by phase interference, while a weighted amplitude constraint algorithm was used to improve convergence. In the noise area, energy efficiency–weighted modulation was introduced to redistribute energy exceeding the threshold energy into nonprocessing areas. This method achieved 95 % optical field uniformity and 73 % energy efficiency in an 80 × 80 pixel pattern, with a speckle contrast as low as 1.5 %. They successfully fabricated complex microstructures with feature sizes ranging from 10–50 μm using TPP, achieving an edge precision of 1.26 μm, as demonstrated in Figure 5b. Zhang et al. [103] applied the aforementioned noise suppression approach to the TPP process. By optimizing the number of overlaid modulating phase masks, they enhanced the uniformity of the structured optical field to nearly 100 % on the focal plane, thereby significantly improving the fabrication quality of micropatterned structures. This method was further applied to fabricating Dammann gratings with various shapes, as displayed in Figure 5c. In addition to improving the fabrication quality of FMNS in static projection fabrication, the multifield noise homogenization method can also be applied to dynamic scanning fabrication to improve the uniformity of the fabricated structure, as displayed in Figure 5d [94].

Table 2 provides a summary of the advantageous effects of FMNS achieved by different phase holographically modulated femtosecond laser technologies.

**Table 2:** Summary of fabrication quality of FMNS achieved by different phase holographically modulated femtosecond laser technologies.

Technologies	Materials	Advantageous effects	FMNS	References
Debye diffraction integration algorithm	Photoresist	Roughness ∼10 nm	3D chiral microstructures	[81]
Patterned exposure with direct laser writing	Photoresist	Roughness <200 nm	Microfluidic devices	[94]
Zernike polynomial	Glass	Aspect ratio: 1.1−1.5	Microhole	[96]
Zernike polynomial + varying input beam ellipticity	PMMA	Aspect ratio: ≤1	Microfluidic channels	[97]
Zernike polynomial + slit beam modulating	Glass	Wavelength polarization sensitivity: 4 pm	FBG	[98]
SSTF	Glass	RMS error: uniform area along the *y* axis is 0.046	Microchannels inside glass	[101]
Improved complex-amplitude modulation	Photoresist, gold film	Uniformity of optical field: 95 %	Micropattern	[102]
MRAF	Photoresist	Uniformity of optical field: ∼100 % on the focal plane	2D microstructures	[103]

## 5 Improvement of fabrication efficiency: parallel fabrication and projection fabrication

### 5.1 Parallel fabrication with multifocal optical fields

**Figure 6:**
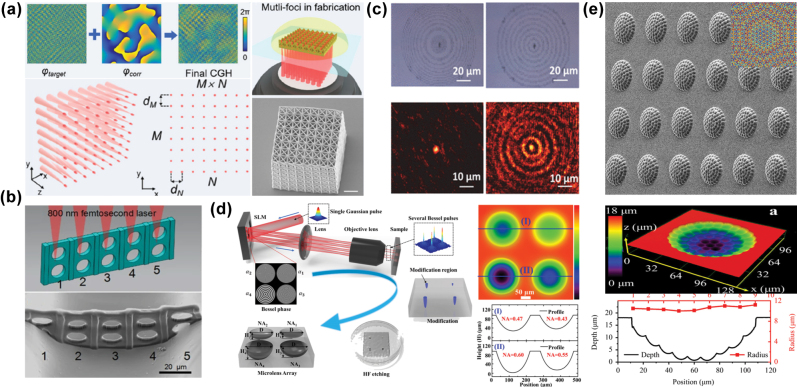
Improvement of fabrication efficiency through projection of multifocal optical fields. (a) Schematic and results of microstructure fabrication using high-speed scanning and high-throughput 3D printing technology based on holographic multifocal points arrays [105]. (b) Schematic and results of microchannel filter sieves fabricated via parallel multifocal points optical field–induced TPP [106]. (c) Fresnel zone plates and Bessel zone plates fabricated via a single-shot projection [107]. (d) Schematic and results of concave lens array fabrication based on axial length differences among focal points within a multifocal points array [108]. (e) Microlens arrays fabricated via multifocal points optical fields with different focal lengths [110]. (a) Is reprinted from Ref. [105], with permission. Copyright 2024 American Chemical Society; (b) is reprinted from Ref. [106], with permission (CC BY 4.0); (c) is reprinted from Ref. [107], with permission. Copyright 2018 American Chemical Society; (d) is reprinted from Ref. [108], with permission (CC BY 4.0); (e) is reprinted from Ref. [110], under the terms of the Open Access Publishing Agreement.

The initial application of phase holography spatial optical field modulation in femtosecond laser fabrication was to transform a single femtosecond laser focal point into an array of equally intense focal spots, enabling parallel fabrication of FMNS structures. In 2005, Hayasaki et al. [104] from Tokushima University, Japan, first realized multifocal points femtosecond laser parallel fabrication based on phase holography modulating using an SLM. By loading a phase hologram computed via the GS algorithm onto the SLM, they successfully fabricated a 1 × 9 array of micro-pits on a glass surface in a single projection. Subsequently, multifocal points optical fields have been widely applied across various fabrication technologies. Zhang et al. [105] proposed a high-throughput 3D printing technology integrating an SLM with a galvanometer scanning system, achieving high-speed scanning of holographic multifocal point arrays. In this method, the target optical field was positioned at the diffraction center of the SLM, and a holographic correction beam was introduced to suppress zero-order diffraction, achieving parallel fabrication with over 400 uniform focal points, as displayed in Figure 6a. Xu et al. [106] used a laterally distributed multifocal points array to induce TPP for the rapid fabrication of filter sieve structures inside microchannels, as displayed in Figure 6b. This technology also allowed precise control of the pore size and density of the sieve by adjusting the number and spacing of focal points, which can be used to sort and collect cells with different sizes. Carstensen et al. [107] used a femtosecond laser to generate a multifocal points array arranged in a Fresnel zone plate and Bessel zone plate pattern. They selectively transformed silver films deposited on silica nanopillar arrays into nanoparticles with distinct resonance spectra by using the femtosecond laser–induced dewetting effect of metal thin films. Consequently, the Fresnel zone plate and Bessel zone plate were successfully fabricated using a single-shot projection, as displayed in Figure 6c.

The use of an SLM not only enables the transformation of a single focal point into an array of focal spots with uniform energy but also allows for precise control over the axial length and energy distribution of individual focal points within the multifocal array. This capability facilitates customized multifocal parallel fabrication, enabling more refined manipulation of the physicochemical properties and structural morphology of the fabricated materials. Liu et al. [108], [109] used variations in axial length among focal points within a multifocal points array to quantitatively control the modified volume inside fused silica. This method can induce various modified structures in a single-shot projection, which were subsequently fabricated using wet etching to form microlens arrays with multiple numerical apertures (NA), as displayed in Figure 6d. Similarly, Chen et al. [110] fabricated a micro-pit array on curved fused silica substrates using a multifocal optical field with hexagonally packed focal spots of varying focal lengths. These micro-pits were subsequently enlarged into microlens arrays using wet etching, as displayed in Figure 6e.

These studies effectively solved the problem of balancing high spatial resolution with an extended depth of field in microlens applications. Furthermore, Silvennoinen et al. [111] used grayscale multifocal points optical field projection to fabricate grayscale patterns composed of micro-pits of varying sizes on the surface of silicon. Compared to binary micro-pit structures, these grayscale-patterned structures provided more intricate pattern details. Based on the principle that reduced graphene oxide (rGO) with different reduction degrees modulates incident laser differently of incident laser, Li et al. [112] fabricated high-order phase holograms on rGO by controlling the focal point’s position and relative energy ratio between focal points in the multifocus points optical field and using a grayscale multifocus points femtosecond laser.

### 5.2 Patterned optical field projection fabrication

**Figure 7:**
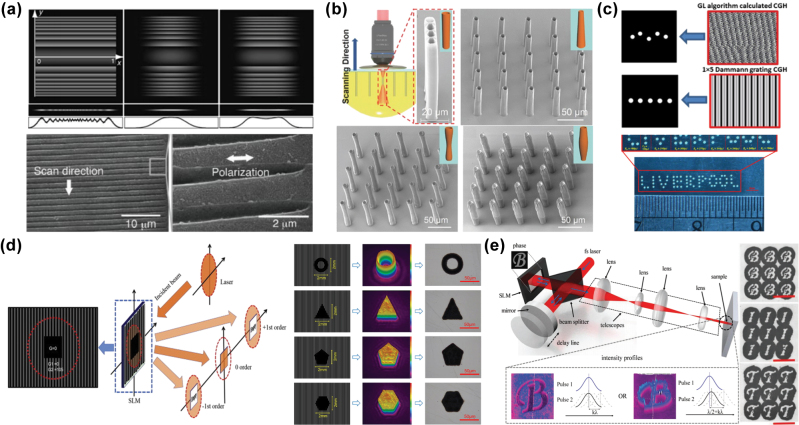
Improvement of fabrication efficiency through projection of patterned optical fields. (a) Improvement of microgroove fabrication efficiency using a line-shaped optical field [113]. (b) Fabrication of microtubules with different cross-sectional shapes using an annular optical field [116]. (c) Rapid drilling of aluminum thin films enabled by an annular optical field [115]. (d) Generation of a patterned optical field by spatially separating the reflected light from the patterned and nonpatterned areas using a grating phase [117]. (e) Modulation of patterned optical fields with arbitrary shapes and intensity distributions based on two-beam interference [118]. (a) Is reprinted from Ref. [113], under the terms of the Open Access Publishing Agreement; (b) is reprinted from Ref. [116], with permission. Copyright 2017 John Wiley and Sons; (c) is reprinted from Ref. [115]. Published by IOP Publishing Ltd. CC BY 4.0; (d) is reprinted from Ref. [117], with permission. Copyright 2018 Elsevier; (e) is reprinted from Ref. [118], with permission, Copyright 2018 John Wiley and Sons.

When the spacing between focal points in a multifocal points optical field is reduced to the size of a single focal spot, the modulated optical field can be regarded as a patterned optical field. Patterned optical fields are widely used in the projection-based fabrication of micropatterned FMNS. Since a single optical field can directly fabricate the desired patterned structure, this approach not only significantly enhances fabrication efficiency but also effectively avoids issues associated with single-focal direct writing. These issues include increased recast layer thickness and an expanded heat-affected zone at the pattern edges, which result from excessive pulse accumulation due to the frequent start-stop motion of the translation stage. Hasegawa et al. [113] used an SLM loaded with a cylindrical lens phase to modulate the focal field of a femtosecond laser into a line-shaped beam, which was then projected onto a glass surface to fabricate periodic microgroove structures. Since each microgroove was fabricated with a single pulse, this method not only improved fabrication efficiency but also eliminated the formation of laser-induced periodic surface structures (LIPSS) at the groove bottom, which typically occurs due to the multipulse incubation effect in single-focal direct writing. Consequently, the surface smoothness at the groove bottom was significantly improved, as displayed in Figure 7a. Except for line-shaped optical fields, annular optical fields of various forms can be generated using vortex phases [114], Bessel phases [115], or a combination of both [116]. These optical fields can be applied in different fabrication scenarios. For instance, in conjunction with TPP, annular optical fields with varying diameters along the optical axis have been used to fabricate microtubules for cellular or drug transport, as displayed in Figure 7b [116]. Additionally, the closed-loop nature of annular optical fields enables rapid drilling of metallic thin films, as demonstrated in Figure 7c [115]. Beyond simple line-shaped optical fields, Liu et al. [117] designed more complex patterned optical fields using a noniterative phase design method. This method involves defining a pattern area at the center of the phase map, overlaying a grating phase onto the nonpattern area to spatially separate the reflected optical fields from the two areas, and finally filtering out the reflected light from the nonpattern area to obtain the desired patterned optical field, as displayed in Figure 7d. Li et al. [118] used the principle of two-beam interference by integrating an SLM as one of the mirrors in a Michelson interferometer. A patterned optical field was generated by modulating a pulse with a pattern-designed phase hologram loaded onto the SLM, followed by interference with an unmodulated pulse reflected from another mirror, as displayed in Figure 7e. Furthermore, this method allows arbitrary modulation of the energy distribution within the patterned optical field, thereby improving energy utilization efficiency.

### 5.3 3D optical field volume projection fabrication

Compared to 2D-shaped optical fields, such as multifocal points array, line-shaped, and planar optical fields, a single 3D optical field enables *in situ* volume projection fabrication of 3D structures within a transparent material, providing a more significant improvement in fabrication efficiency. The simplest form of a 3D optical field is an axially elongated optical field, represented by Bessel beams and their variations (i.e., Bessel-like beams). Bessel beams are primarily used for fabricating high-aspect-ratio microhole structures. Compared to varying the parameters of axicon lenses, generating Bessel beams using an SLM provides greater flexibility, as the intensity distribution of the modulated optical field can be controlled by adjusting the phase hologram.

**Figure 8:**
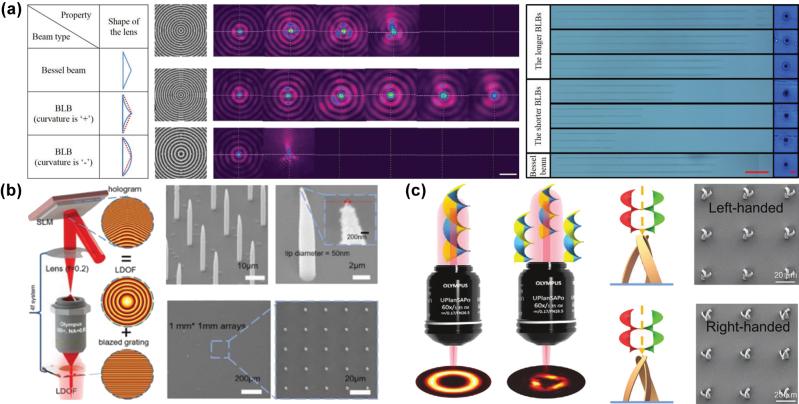
Improvement of fabrication efficiency through 3D optical fields volume projection fabrication. (a) Fabrication of microhole structures with varying depth-to-diameter ratios using Bessel-like beams with adjustable nondiffractive regions [119]. (b) High-aspect-ratio micropillar structures fabricated using an axially elongated optical field [121]. (c) 3D chiral microstructures fabricated based on multiple parallel vortex optical field [81]. (a) Is reprinted from Ref. [119], under the terms of the Open Access Publishing Agreement; (b) is reprinted with permission from Ref [121] © Optica Publishing Group; (c) is reprinted from Ref. [81], with permission. Copyright 2022 John Wiley and Sons.

Yao et al. [119] continuously tuned the nondiffractive region length of Bessel beams by overlaying focusing or diffusing phases onto a zero-order Bessel phase, as displayed in Figure 8a. This method enables the fabrication of microholes with different aspect ratios without moving the sample, simply by modifying the computational parameters of the phase hologram [120]. Furthermore, in additive manufacturing technologies such as femtosecond laser–induced TPP or multiphoton polymerization (MPP), Bessel beams and other axially elongated optical fields allowed for *in situ* projection fabrication of high-aspect-ratio micropillar structures, as displayed in Figure 8b [121]. Wu et al. [81] utilized the interference of multiple parallel vortex beams to generate 3D chiral microstructures, leveraging capillary forces for subunit assembly of these structures, as demonstrated in Figure 8c. The fabrication efficiency was enhanced by more than 100 times.

**Figure 9:**
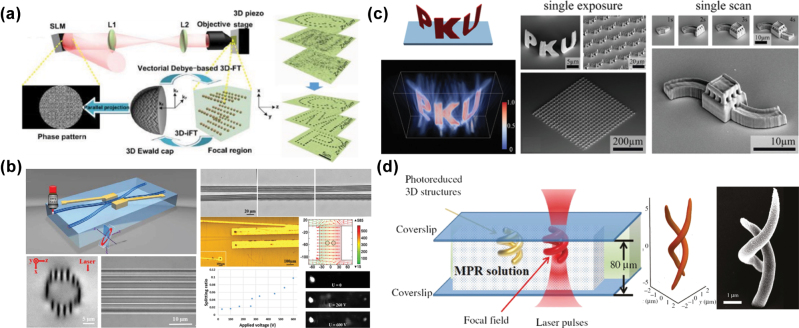
Improvement of fabrication efficiency through 3D multifocal optical fields and arbitrarily shaped volumetric optical fields projection fabrication. (a) Schematic of a uniformly patterned lattice structure fabricated at different depths using a 3D Fourier transform iterative algorithm based on vector Debye diffraction [123]. (b) Type-III optical waveguide structures and waveguide coupling devices fabricated in a single scan using a 3D annular multifocal points optical field [124]. (c) 3D structures fabricated via TPP using either volumetric projection or serial stitching of a 3D optical field [125]. (d) Metallic helical chiral structures fabricated using a dual-helical beam based on the MPP effect [126]. (a) Is reprinted with permission from Ref [123] © Optica Publishing Group; (b) is reprinted from Ref. [124], with permission from Photonics Research; (c) is reprinted from Ref. [125], with permission. Copyright 2019 John Wiley and Sons; (d) is reprinted from Ref. [126], with permission (CC BY 4.0).

For 3D optical fields with more complex intensity distributions, such as 3D multifocal points optical fields or arbitrarily shaped volumetric optical fields, phase hologram iterative algorithms suitable for 3D optical field modulation are required, as their phase holograms lack fixed analytical functions. Jesacher et al. [122] used a 3D GS algorithm to generate a phase hologram for a 3D multifocal points optical field with a like-crystalline distribution. They successfully fabricated uniform 3D cavity structure arrays inside lithium niobate and fused silica by introducing spherical aberration compensation. Ren et al. [123] improved the uniformity of focal intensities among different focal point arrays along the optical axis by improving the 2D Fourier iterative algorithm with a 3D Fourier transform based on vector Debye diffraction. This method facilitated the fabrication of uniformly sized patterned lattice structures at different depths within the sample, as displayed in Figure 9a. Zhang et al. [124] applied the Yang–Gu algorithm to generate a 3D annular multifocal optical field, allowing the fabrication of Type-III optical waveguide structures and waveguide coupling devices within lithium niobate through a single scan, as displayed in Figure 9b. Furthermore, based on the electro-optic refractive index of lithium niobate crystal, signal coupling between different optical waveguides and the adjustment of spectral coefficients were achieved by applying a voltage to change the refractive index of the material.

Furthermore, 3D optical fields with complex intensity distributions can also be applied in additive manufacturing technology, including femtosecond laser-induced TPP, MPP, and metal ion reduction. Yang et al. [125] used the nonlinear effects of TPP to fabricate high-quality 3D structures by improving iterative algorithms and optimizing the energy distribution of the 3D optical field, as displayed in Figure 9c. For small-scale structures, a single modulated optical field was sufficient for projection fabrication. However, for large and complex structures, a 3D object could be longitudinally sliced to generate a series of 2D patterns and corresponding phase holograms, which were subsequently stitched together to fabricate intricate 3D structures. Liu et al. [126] first modulated a femtosecond laser beam into a dual-helical structured optical field and subsequently employed the MPP effect to reduce silver ions in a silver nitrate solution into silver nanoparticles. These nanoparticles were then self-assembled into a metallic dual-helical structure following the distribution of the structured optical field, as displayed in Figure 9d. The fabricated dual-helical structures exhibited low surface roughness and high reproducibility because the heat accumulation caused by pulse overlap was reduced, making this method suitable for the fabrication of chiral optical devices in the mid-infrared spectral range. Although 3D-shaped optical fields significantly improve the fabrication efficiency of femtosecond lasers, their current applications are primarily focused on structural formation. Research on the application of 3D optical field modulation in fabricating micro–nano optical devices is still in its early stages.

Table 3 provides a summary of the fabrication efficiency of FMNS achieved by different phase holographically modulated femtosecond laser technologies.

**Table 3:** Summary of fabrication efficiency of FMNS achieved by different phase holographically modulated femtosecond laser technologies.

Technologies	Materials	Fabrication rate	FMNS	References
PPLL	Cr film and Au film	50,000 structures/min	Concentric ring	[90]
Patterned exposure with direct laser writing	Photoresist	Improved ∼20 times compared with conventional TPP	Microfluidic devices	[94]
MRAF	Photoresist	167.5 voxels/s	Microtraps	[103]
Galvanometric scanner + multifocus array	Photoresist	1.49 × 10^8^ voxels/s	Dot arrays	[105]
Multifoci parallel microfabrication	Photoresist	Improved 9 times compared with conventional TPP	Microdevices in microfluidic chip	[106]
Fs-laser parallel fabrication	Silica	Improved 4 times compared with a conventional method	Microlens	[108]
Multifoci parallel microfabrication	Silica	Improved 61 times compared with conventional method	Microlens	[110]
Multifoci parallel microfabrication	Silicon	Improved 400 times compared with a conventional method	Ablation crater	[111]
Line-shaped fs laser direct writing	Glass, ITO, stainless steel	Improved 400 times compared with Gaussian beam	Microgroove	[113]
Multifoci parallel microfabrication	Photoresist	100 ms	Microtube	[114]
Multiple annular beams	Stainless steel	Improved 400 times compared with single circular beam	Microhole	[115]
Axial scanning of the ring-structure Bessel beams + dynamic display of holograms	Photoresist	1 microtube/5 s, (conventional method need tens of minutes)	Microtube	[116]
Spatiotemporal-interference–based fs laser	Silicon gold film	10 patterns/s	Micropattern	[118]
Axially elongated optical field	Glass	10 modified microchannel/1 s	Modified microchannel	[120]
Debye diffraction integration algorithm	Photoresist	Improved 102 times compared with conventional method	3D chiral microstructures	[81]
Axially elongated optical field	Photoresist	Improved 100 times compared with conventional method	Micropillar	[121]
3D annular multifocal optical field	Lithium niobate	Improved 16 times compared with conventional method	Optical waveguide	[124]
3D pattern optical field	Photoresist	0.1 s	3D microstructure	[125]
Double-helix optical field	Silver	Improved 20 times compared with conventional method	Double helix metamaterial	[126]

## 6 Conclusions

Based on phase holography modulated femtosecond laser fabrication technology, various types of spatial optical fields can be generated by loading phase holograms onto an SLM, enabling adaptation to different fabricating requirements. This approach significantly improves the flexibility and applicability of femtosecond laser fabrication, providing an essential solution for achieving high precision, high quality, and high efficiency in femtosecond laser fabrication. In terms of improving the fabrication resolution, the technologies achieve the feature size fabrication exceeding the diffraction limit on metals, semiconductors, polymers, and other materials through strategies such as dual-focus light field induced material phase transition and multimode light field cooperative regulation. Regarding optimization of fabrication quality, technologies such as spherical aberration compensation phase algorithms, spatiotemporal synchronized focusing, and noise suppression algorithms have substantially improved the uniformity of FMNS. For improving fabrication efficiency, multifocal points parallel fabrication, patterned exposure, and volumetric 3D projection technologies have successfully reduced the fabrication time of complex structures from several hours to mere minutes.

However, the development of phase holographic modulation–based femtosecond laser technology still faces several unresolved challenges: (1) Although pure phase modulators offer higher energy efficiency compared to amplitude modulation devices, current hardware systems still fall short in terms of pixel resolution and modulation accuracy. These limit the spatial fidelity and precision of the modulated light fields and need to be addressed through further advancements in device fabrication and control technologies. (2) In practical applications, real-time and continuous updating of phase holograms is often required. The refresh rate of currently available SLM is limited to several kHz, which restricts the system’s dynamic response. Improving the refresh rate to tens or even hundreds of kHz would greatly expand the application scope and efficiency of phase holographically modulated femtosecond laser technology. (3) The generation and modulation of complex structured optical fields remain an area of ongoing research. At present, the fabricated structures are largely limited to simple and regular patterns. It is, therefore, essential to further develop theoretical frameworks and modulation strategies that enable the efficient generation of high-quality, application-specific structured light fields. This includes controlling amplitude, phase, and polarization simultaneously, and leveraging technology such as direct superposition, spatial tailoring, and optical interference to achieve customizable and high-fidelity optical field distributions.

In summary, phase holography modulated femtosecond laser has demonstrated significant potential in FMNS fabrication. With further exploration and improvement of its applications, it is expected to facilitate the integration of diverse micro–nano devices and promote its industrialization.

## References

[1] Monticone F. (2023). Toward ultrathin optics. *Science*.

[2] Liu S. F. (2022). 3D nanoprinting of semiconductor quantum dots by photoexcitation-induced chemical bonding. *Science*.

[3] Overvig A., Mann S. A., Alù A. (2024). Spatio-temporal coupled mode theory for nonlocal metasurfaces. *Light Sci. Appl.*.

[4] Wang H. (2024). Two-photon polymerization lithography for imaging optics. *Int. J. Extrem. Manuf.*.

[5] Miller D. (2023). Why optics needs thickness. *Science*.

[6] Shastri K., Monticone F. (2023). Nonlocal flat optics. *Nat. Photonics*.

[7] Hu J., Zhu S., Lv Y., Guo R., Gu M., Zhang Y. (2024). Ultrathin, wavelength-multiplexed and integrated holograms and optical neural networks based on 2D perovskite nanofilms. *Laser Photon. Rev.*.

[8] Ye Y. (2025). Investigation of the modification intensity and distribution in silica glass via ultrafast laser direct writing. *Appl. Opt.*.

[9] Wang H. (2023). Two-photon polymerization lithography for optics and photonics: fundamentals, materials, technologies, and applications. *Adv. Funct. Mater.*.

[10] Thompson A. J., Power M., Yang G. Z. (2018). Micro-scale fiber-optic force sensor fabricated using direct laser writing and calibrated using machine learning. *Opt. Express*.

[11] Li M. (2024). Wide-size range and high robustness self-assembly micropillars for capturing microspheres. *ACS Appl. Mater. Interfaces*.

[12] Zhang W. (2021). Structural multi-colour invisible inks with submicron 4D printing of shape memory polymers. *Nat. Commun.*.

[13] Martella D., Nocentini S., Nuzhdin D., Parmeggiani C., Wiersma D. S. (2017). Photonic microhand with autonomous action. *Adv. Mater.*.

[14] Yi P., Fu X., Liu Y., Zhang X., Zhang C., Li X. (2023). Triboelectric active pressure sensor with ultrabroad linearity range by femtosecond laser shaping based on electrons dynamics control. *Nano Energy*.

[15] Cui X., Nie J., Zhang Y. (2024). Recent advances in high charge density triboelectric nanogenerators. *Int. J. Extrem. Manuf.*.

[16] Yi P. (2025). Contact mode controls droplet generate electricity by femtosecond laser. *Nano Energy*.

[17] Zhang T. (2021). Surface-microengineering for high-performance triboelectric tactile sensor via dynamically assembled ferrofluid template. *Nano Energy*.

[18] Yao G. (2020). Bioinspired triboelectric nanogenerators as self-powered electronic skin for robotic tactile sensing. *Adv. Funct. Mater.*.

[19] Cao Y. (2022). Highly sensitive self-powered pressure and strain sensor based on crumpled MXene film for wireless human motion detection. *Nano Energy*.

[20] Chen J., Low Z. X., Feng S., Zhong Z., Xing W., Wang H. (2021). Nanoarchitectonics for electrospun membranes with asymmetric wettability. *ACS Appl. Mater. Interfaces*.

[21] Ma Z. (2023). Lotus leaf inspired sustainable and multifunctional Janus film for food packaging. *Chem. Eng. J.*.

[22] Liang Z. (2023). Aluminum-based heterogeneous surface for efficient solar desalination and fog harvesting processed by a picosecond laser. *ACS Appl. Mater. Interfaces*.

[23] Yue P., Zhang M., Zhao T., Liu P., Peng F., Yang L. (2024). Eco-friendly epoxidized Eucommia ulmoides gum based composite coating with enhanced super-hydrophobicity and corrosion resistance properties. *Ind. Crops Prod.*.

[24] Huang K., Si Y., Hu J. (2024). Fluid unidirectional transport induced by structure and ambient elements across porous materials: from principles to applications. *Adv. Mater.*.

[25] Hassan M. M. (2019). Au@Ag nanostructure based SERS substrate for simultaneous determination of pesticides residue in tea via solid phase extraction coupled multivariate calibration. *Lwt*.

[26] Xu Y. (2020). Mesoporous silica supported orderly-spaced gold nanoparticles SERS-based sensor for pesticides detection in food. *Food Chem.*.

[27] Liao C. (2019). High-speed all-optical modulator based on a polymer nanofiber Bragg grating printed by femtosecond laser. *ACS Appl. Mater. Interfaces*.

[28] Sun Y. (2021). Facile fabrication of three-dimensional gold nanodendrites decorated by silver nanoparticles as hybrid SERS-active substrate for the detection of food contaminants. *Food Control*.

[29] Zhu A., Xu Y., Ali S., Ouyang Q., Chen Q. (2021). Au@Ag nanoflowers based SERS coupled chemometric algorithms for determination of organochlorine pesticides in milk. *Lwt*.

[30] Li H., Hassan M. M., He Z., Haruna S. A., Chen Q., Ding Z. (2022). A sensitive silver nanoflower-based SERS sensor coupled novel chemometric models for simultaneous detection of chlorpyrifos and carbendazim in food. *Lwt*.

[31] Wu D. (2009). 100% fill-factor aspheric microlens arrays (AMLA) with sub-20-nm precision. *IEEE Photonics Technol. Lett.*.

[32] Luo Z., Wang C., Yin K., Dong X., Chu D., Duan J. (2016). Rapid fabrication of cylindrical microlens array by shaped femtosecond laser direct writing. *Appl. Phys. A*.

[33] Han W., Jiang L., Li X. W., Liu Y., Lu Y. (2015). Femtosecond laser induced tunable surface transformations on (111) Si aided by square grids diffraction. *Appl. Phys. Lett.*.

[34] Zuo P. (2024). Controllable fabrication of hydrophilic surface micro/nanostructures of CFRP by femtosecond laser. *ACS Omega*.

[35] Sugioka K. (2017). Progress in ultrafast laser processing and future prospects. *Nanophotonics*.

[36] Liu Y. (2024). Controllable Si micro-structuring by temporally modulated single-shot femtosecond pulse lithography. *Appl. Phys. Lett.*.

[37] Zhang Y., Pei J., Huang Z., Jiang L., Yin K., Jiang J. (2024). Maskless femtosecond-laser-processed ionotronic double-gate transistor array for pattern adaptation emulation. *Adv. Funct. Mater.*.

[38] Yang Q. X., Liu H. L., He S., Tian Q., Xu B., Wu P. (2021). Circular cladding waveguides in Pr: YAG fabricated by femtosecond laser inscription: Raman, luminescence properties and guiding performance. *Opto-Electron. Adv.*.

[39] Chen L. (2021). Large-area straight, regular periodic surface structures produced on fused silica by the interference of two femtosecond laser beams through cylindrical lens. *Opto-Electron. Adv.*.

[40] Livakas N., Skoulas E., Stratakis E. (2020). Omnidirectional iridescence via cylindrically-polarized femtosecond laser processing. *Opto-Electron. Adv.*.

[41] Jia Y. C., Wang S. X., Chen F. (2020). Femtosecond laser direct writing of flexibly configured waveguide geometries in optical crystals: fabrication and application. *Opto-Electron. Adv.*.

[42] Liu Y., Li X., Huang J. (2022). High-uniformity submicron gratings with tunable periods fabricated through femtosecond laser-assisted molding technology for deformation detection. *ACS Appl. Mater. Interfaces*.

[43] Jiang L., Wang A. D., Li B., Cui T. H., Lu Y. F. (2018). Electrons dynamics control by shaping femtosecond laser pulses in micro/nanofabrication: modeling, method, measurement, and application. *Light Sci. Appl.*.

[44] Liu Y. (2023). Femtosecond laser printing-assisted etching tailored hard and brittle micro-convex surface. *Opt. Lett.*.

[45] Gattass R. R., Mazur E. (2008). Femtosecond laser micromachining in transparent materials. *Nat. Photonics*.

[46] Ji X. (2014). Polarization-dependent elliptical crater morphologies formed on a silicon surface by single-shot femtosecond laser ablation. *Appl. Opt.*.

[47] Li Z. Z. (2020). O-FIB: far-field-induced near-field breakdown for direct nanowriting in an atmospheric environment. *Light Sci. Appl.*.

[48] Sugioka K., Cheng Y. (2014). Ultrafast lasers-reliable tools for advanced materials processing. *Light Sci. Appl.*.

[49] Paz V. F. (2012). Development of functional sub-100 nm structures with 3D two-photon polymerization technique and optical methods for characterization. *J. Laser Appl.*.

[50] Li T. (2024). Reconfigurable hologram response to liquid via the femtosecond laser direct writing of 3D micropillars. *Adv. Opt. Mater.*.

[51] Hu Q. (2017). Additive manufacture of complex 3D Au-containing nanocomposites by simultaneous two-photon polymerization and photoreduction. *Sci. Rep.*.

[52] Yang X. (2022). Optimization mechanism and applications of ultrafast laser machining towards highly designable 3D micro/nano structuring. *RSC Adv.*.

[53] Tielen V., Bellouard Y. (2014). Three-dimensional glass monolithic micro-flexure fabricated by femtosecond laser exposure and chemical etching. *Micromachines*.

[54] Wu M. N. (2023). Microheater-integrated microlens array for robust rapid fog removal. *ACS Appl. Mater. Interfaces*.

[55] Hua J. G. (2023). Laser-induced cavitation-assisted true 3D nano-sculpturing of hard materials. *Small*.

[56] Peng D. (2024). Evaluation of a novel femtosecond laser ablation system for in situ analysis based on two-dimensional galvanometer scanners. *Anal. Chem.*.

[57] Wlodarczyk K. L., Ardron M., Weston N. J., Hand D. P. (2019). Holographic watermarks and steganographic markings for combating the counterfeiting practices of high-value metal products. *J. Mater. Process. Technol.*.

[58] Low M. J. (2020). Laser-induced reduced-graphene-oxide micro-optics patterned by femtosecond laser direct writing. *Appl. Surf. Sci.*.

[59] Zhang F., Duan J. A., Zhou X. F., Wang C. (2018). Broadband and wide-angle antireflective subwavelength microstructures on zinc sulfide fabricated by femtosecond laser parallel multi-beam. *Opt. Express*.

[60] Nakata Y., Yoshida M., Osawa K., Miyanaga N. (2017). Fabricating a regular hexagonal lattice structure by the interference pattern of six femtosecond laser beams. *Appl. Surf. Sci.*.

[61] Wu H. (2019). Large area metal micro-/nano-groove arrays with structural color and anisotropic wetting are fabricated by one-step focused laser interference lithography. *Nanoscale*.

[62] Salter P. S., Booth M. J. (2019). Adaptive optics in laser processing. *Light Sci. Appl.*.

[63] Kang M., Han C., Jeon H. (2020). Submicrometer-scale pattern generation via maskless digital photolithography. *Optica*.

[64] Englesbe A. C., He Z., Nees J. A., Thomas A. G., Schmitt-Sody A., Krushelnick K. (2016). Control of the configuration of multiple femtosecond filaments in air by adaptive wavefront manipulation. *Opt. Express*.

[65] Zhang Z., You Z., Chu D. (2014). Fundamentals of phase-only liquid crystal on silicon (LCOS) devices. *Light Sci. Appl.*.

[66] Wang Z. P. (2022). High efficiency and scalable fabrication of Fresnel zone plates using holographic femtosecond pulses. *Nanophotonics*.

[67] Stanley M. (2004). 3D electronic holography display system using a 100 mega-pixel spatial light modulator. *Proc. SPIE*.

[68] Schonbrun E. (2005). 3D interferometric optical tweezers using a single spatial light modulator. *Opt. Express*.

[69] Fürhapter S., Jesacher A., Bernet S., Ritsch-Marte M. (2005). Spiral phase contrast imaging in microscopy. *Opt. Express*.

[70] Zhang C. (2014). An improved multi-exposure approach for high quality holographic femtosecond laser patterning. *Appl. Phys. Lett.*.

[71] Gerchberg R. W. (1972). A practical algorithm for the determination of phase from image and diffraction plane pictures. *Optik*.

[72] Wu Y., Wang J., Chen C., Liu C. J., Jin F. M. (2021). Adaptive weighted Gerchberg Saxton algorithm for generation of phase-only hologram with artifacts suppression. *Opt. Express*.

[73] Alsaka D. Y., Arpali Ç., Arpali S. A. (2018). A comparison of iterative Fourier transform algorithms for image quality estimation. *Opt. Rev.*.

[74] Wang J. (2022). Three-dimensional holographic femtosecond laser parallel processing method with the fractional Fourier transform for glass substrates. *Ceram. Int.*.

[75] Dufresne E. R., Spalding G. C., Dearing M. T., Sheets S. A., Grier D. G. (2001). Computer generated holographic optical tweezer arrays. *Rev. Sci. Instrum.*.

[76] Kirkpatrick S., Gelatt C. D., Vecchi M. P. (1983). Optimization by simulated annealing. *Science*.

[77] Rakheja P., Yadav S., Tobria A. (2022). A novel image encryption mechanism based on umbrella map and Yang-Gu algorithm. *Optik*.

[78] Zhu L. W., Sun M. Y., Zhang D. W., Yu J., Wen J., Chen J. (2015). Multifocal array with controllable polarization in each focal spot. *Opt. Express*.

[79] Liu Z. H. (2022). Generation and modulation of controllable multi-focus array based on phase segmentation. *Micromachines*.

[80] Li J. Q., Yan J. F., Jiang L., Yu J., Guo H., Qu L. (2023). Nanoscale multi-beam lithography of photonic crystals with ultrafast laser. *Light Sci. Appl.*.

[81] Pan D. (2022). Rapid fabrication of 3D chiral microstructures by single exposure of interfered femtosecond vortex beams and capillary-force-assisted self-assembly. *Adv. Funct. Mater.*.

[82] Guan J. (2018). Non-iterative dartboard phase filter for achieving multifocal arrays by cylindrical vector beams. *Opt. Express*.

[83] Hasegawa S., Hayasaki Y. (2021). Femtosecond laser processing with adaptive optics based on convolutional neural network. *Opt. Laser Eng.*.

[84] Buske P., Völl A., Eisebitt M., Stollenwerk J., Holly C. (2022). Advanced beam shaping for laser materials processing based on diffractive neural networks. *Opt. Express*.

[85] Huang J., Xu K., Xu S. (2025). Super-resolution laser machining. *Int. J. Mach. Tools Manuf.*.

[86] Wang A. D. (2015). Mask-free patterning of high-conductivity metal nanowires in open air by spatially modulated femtosecond laser pulses. *Adv. Mater.*.

[87] Xu Z. (2019). Flash ablation of tunable and deep-subwavelength nanogap by using a spatially modulated femtosecond laser pulse for plasmonic application. *ACS Appl. Nano Mater.*.

[88] Zhou S. (2021). Fabrication of nanogap structures through spatially shaped femtosecond laser modification with the assistance of wet chemical etching. *Opt. Lett.*.

[89] Zhao L. (2022). Far-field parallel direct writing of sub-diffraction-limit metallic nanowires by spatially modulated femtosecond vector beam. *Adv. Mater. Technol.*.

[90] Qiu P., Yuan D., Huang J., Li J., Xu S. (2024). Polarization-modulated patterned laser sculpturing of optical functional hierarchical micro/nanostructures. *Adv. Opt. Mater.*.

[91] Huang L., Xu K., Yuan D., Hu J., Wang X., Xu S. (2022). Sub-wavelength patterned pulse laser lithography for efficient fabrication of large-area metasurfaces. *Nat. Commun.*.

[92] Hasegawa S. (2019). Spatial phase shaping of ultrashort laser pulses to overcome the diffraction limit. *OSA Continuum*.

[93] Wang Z. (2021). Thermally reconfigurable hologram fabricated by spatially modulated femtosecond pulses on a heat-shrinkable shape memory polymer for holographic multiplexing. *ACS Appl. Mater. Interfaces*.

[94] Zhang C. (2020). Rapid fabrication of high-resolution multi-scale microfluidic devices based on the scanning of patterned femtosecond laser. *Opt. Lett.*.

[95] Cumming B. P., Turner M. D., Schröder-Turk G. E., Debbarma S., Luther-Davies B., Gu M. (2014). Adaptive optics enhanced direct laser writing of high refractive index gyroid photonic crystals in chalcogenide glass. *Opt. Express*.

[96] Kontenis G., Gailevičius D., Jonušauskas L., Purlys V. (2020). Dynamic aberration correction via spatial light modulator (SLM) for femtosecond direct laser writing: towards spherical voxels. *Opt. Express*.

[97] Roth G. L., Rung S., Esen C., Hellmann R. (2020). Microchannels inside bulk PMMA generated by femtosecond laser using adaptive beam shaping. *Opt. Express*.

[98] Salter P. S., Woolley M. J., Morris S. M., Booth M. J., Fells J. A. J. (2018). Femtosecond fiber Bragg grating fabrication with adaptive optics aberration compensation. *Opt. Lett.*.

[99] Wang Z. (2020). Three-dimensional microwave holography based on broadband Huygens’ metasurface. *Phys. Rev. Appl.*.

[100] Chen L., Zhang H., He Z., Wang X., Cao L., Jin G. (2020). Weighted constraint iterative algorithm for phase hologram generation. *Appl. Sci.*.

[101] Sun B. (2018). Four-dimensional light shaping: Manipulating ultrafast spatiotemporal foci in space and time. *Light Sci. Appl.*.

[102] Li T. (2024). High-quality micropattern printing by complex-amplitude modulation holographic femtosecond laser. *Adv. Opt. Mater.*.

[103] Zhang C. C. (2016). Optimized holographic femtosecond laser patterning method towards rapid integration of high-quality functional devices in microchannels. *Sci. Rep.*.

[104] Hayasaki Y., Sugimoto T., Takita A., Nishida N. (2005). Variable holographic femtosecond laser processing by use of a spatial light modulator. *Appl. Phys. Lett.*.

[105] Zhang L. (2024). High-throughput two-photon 3D printing enabled by holographic multi-foci high-speed scanning. *Nano Lett.*.

[106] Xu B. (2016). High efficiency integration of three-dimensional functional microdevices inside a microfluidic chip by using femtosecond laser multifoci parallel microfabrication. *Sci. Rep.*.

[107] Carstensen M. S., Zhu X. L., Iyore O. E., Mortensen N. A., Levy U., Kristensen A. (2018). Holographic resonant laser printing of metasurfaces using plasmonic template. *ACS Photonics*.

[108] Liu Y. (2022). Morphology adjustable microlens array fabricated by single spatially modulated femtosecond pulse. *Nanophotonics*.

[109] Liu Y. (2023). Single-pixel-adjustable structural color fabricated using a spatially modulated femtosecond laser. *ACS Appl. Mater. Interfaces*.

[110] Wang L., Gong W., Cao X. W., Yu Y. H., Juodkazis S., Chen Q. D. (2023). Holographic laser fabrication of 3D artificial compound μ-eyes. *Light: Adv. Manuf.*.

[111] Silvennoinen M., Kaakkunen J., Paivasaari K., Vahimaa P. (2014). Parallel femtosecond laser ablation with individually controlled intensity. *Opt. Express*.

[112] Li X. P. (2015). Athermally photoreduced graphene oxides for three-dimensional holographic images. *Nat. Commun.*.

[113] Hasegawa S., Shiono K., Hayasaki Y. (2015). Femtosecond laser processing with a holographic line-shaped beam. *Opt. Express*.

[114] Yang L. (2019). Targeted single-cell therapeutics with magnetic tubular micromotor by one-step exposure of structured femtosecond optical vortices. *Adv. Funct. Mater.*.

[115] Kuang Z., Perrie W., Edwardson S. P., Fearon E., Dearden G. (2014). Ultrafast laser parallel microdrilling using multiple annular beams generated by a spatial light modulator. *J. Phys. D: Appl. Phys.*.

[116] Ji S. Y. (2017). Dimension-controllable microtube arrays by dynamic holographic processing as 3D yeast culture scaffolds for asymmetrical growth regulation. *Small*.

[117] Liu D. (2018). Dynamic laser beam shaping for material processing using hybrid holograms. *Opt. Laser Technol.*.

[118] Li B. H. (2018). Flexible gray-scale surface patterning through spatiotemporal-interference-based femtosecond laser shaping. *Adv. Opt. Mater.*.

[119] Yao Z. L. (2018). Non-diffraction-length, tunable, Bessel-like beams generation by spatially shaping a femtosecond laser beam for high-aspect-ratio micro-hole drilling. *Opt. Express*.

[120] Yao Z. (2021). High-efficiency fabrication of computer-generated holograms in silica glass using a femtosecond Bessel beam. *Opt. Laser Technol.*.

[121] Pan D. (2020). Efficient fabrication of a high-aspect-ratio AFM tip by one-step exposure of a long focal depth holographic femtosecond axilens beam. *Opt. Lett.*.

[122] Jesacher A., Booth M. J. (2010). Parallel direct laser writing in three dimensions with spatially dependent aberration correction. *Opt. Express*.

[123] Ren H., Lin H., Li X., Gu M. (2014). Three-dimensional parallel recording with a Debye diffraction-limited and aberration-free volumetric multifocal array. *Opt. Lett.*.

[124] Zhang Q. (2019). Reconfigurable directional coupler in lithium niobate crystal fabricated by three-dimensional femtosecond laser focal field engineering. *Photonics Res*.

[125] Yang D., Liu L., Gong Q., Li Y. (2019). Rapid two-photon polymerization of an arbitrary 3D microstructure with 3D focal field engineering. *Macromol. Rapid Commun.*.

[126] Liu L., Yang D., Wan W., Yang H., Gong Q., Li Y. (2019). Fast fabrication of silver helical metamaterial with single-exposure femtosecond laser photoreduction. *Nanophotonics*.

